# Improving adherence in mental health service users with severe mental illness in South Africa: a pilot randomized controlled trial of a treatment partner and text message intervention vs. treatment as usual

**DOI:** 10.1186/s13104-017-2915-z

**Published:** 2017-11-09

**Authors:** Goodman Sibeko, Henk Temmingh, Sumaya Mall, Peter Williams-Ashman, Graham Thornicroft, Ezra S. Susser, Crick Lund, Dan J. Stein, Peter D. Milligan

**Affiliations:** 1 Department of Psychiatry and Mental Health, University of Cape Town, Cape Town, South Africa; 2 Institute of Psychiatry Psychology and Neuroscience, King’s College London, London, UK; 3 Columbia University Mailman School of Public Health, New York State Psychiatric Institute, New York, USA

**Keywords:** Mental health, Task-shifting, Treatment partner, Adherence, Text message, Mobile health

## Abstract

**Objectives:**

Medication non-adherence is a significant problem in treatment of severe mental disorders and is associated with poor clinical outcomes and increased demand on services. Task-shifting interventions incorporating mobile health may improve adherence in mental health service users in low- and middle-income countries. Seventy-seven participants were recruited from a psychiatric hospital in Cape Town, with 42 randomized to receive the intervention and 35 to treatment as usual. Intervention pairs underwent treatment-partner contracting and psychoeducation, and received monthly text message reminders of clinic appointments. Primary outcomes were intervention acceptability and feasibility. Secondary outcome for efficacy were adherence to clinic visit; relapse; quality of life; symptomatic relief and medication adherence.

**Results:**

Treatment partner and psychoeducation components were acceptable and feasible. The text message component was acceptable but not feasible in its current form. Efficacy outcomes favoured the intervention but did not reach statistical significance. A treatment-partner intervention is acceptable and feasible in a low- and middle-income setting. Work is needed to ensure that additional components of such interventions are tailored to the local context. Appropriately powered efficacy studies are needed.

*Trial Registration* PACTR PACTR201610001830190, Registered 21 October 2016 (Retrospectively registered)

**Electronic supplementary material:**

The online version of this article (10.1186/s13104-017-2915-z) contains supplementary material, which is available to authorized users.

## Introduction

Poor medication adherence is a major problem in the treatment of severe mental disorders and is associated with poor clinical outcomes and increased demand on services [1–3]. Thus, there is significant interest in developing interventions to improve medication adherence in this group [4, 5].

Mental health services are under-resourced globally, with low- and middle-income countries (LMIC) facing a particular challenge [6]. One approach that seeks to address the shortfall is task-shifting, which refers to the delivery of evidence-based interventions by non-specialist workers [7–9]. Treatment partner interventions may represent a useful task-shifting approach in adherence-promotion for mental health service users (MHSU). A MHSU is an individual accessing care, treatment and rehabilitation services via a health establishment for the purpose of enhancing his or her mental health status [10].

Telephone prompts have shown promise in encouraging treatment adherence and clinic attendance [4, 11]. Mobile health (m-health), defined as medical and public health practice supported by mobile devices, is gaining popularity worldwide [12, 13]. While m-health approaches require further investigation to establish efficacy, they have been adopted in maternal and child health, and chronic diseases [12, 14, 15]. This technology has not been widely used in adherence-focused mental health interventions in LMIC.

Approaches to adherence-support have emphasized the importance of health literacy, problem-solving, and social support, as well as the potential of telephone prompts [4, 5]. It is however unclear whether an approach incorporating these components is feasible in a LMIC setting.

Our two-arm non-blinded prospective pilot randomized controlled trial evaluated the acceptability and feasibility of a treatment partner and text message intervention in supporting adherence in people with severe mental illness.

## Main text

### Methods

We recruited over 2 years from Valkenberg hospital (VBH), which offers public psychiatric services in Cape Town, and at 15 psychiatry clinics in the hospital’s catchment area. VBH has 116 male, and 84 female beds with majority of patients having a diagnosis of severe mental illness, constituting a significant service and disability burden [16]. The psychiatry clinics providing post-discharge care in the VBH catchment area are run by mental health nurses.

We screened clinical folders of MHSU in the pre-discharge wards at VBH. We included MHSU diagnosed with schizophrenia spectrum disorder, substance induced psychotic disorder, and bipolar mood disorder type I. We approached eligible MHSU and obtained informed consent from those willing to participate after being informed about the study. Exclusion criteria included: (a) a diagnosis of psychotic disorder due to a general medical condition, dementia, moderate to severe intellectual disability; or (b) suicidality; or (c) an inability to give informed consent.

Participants were randomized to either intervention or treatment as usual (TAU), using a randomization sequence generated by an external statistician. We ensured allocation concealment by using opaque, sealed sequentially numbered envelopes.

Intervention participants nominated a treatment partner from within their social support network. An appointment was set at a date preceding the participant’s discharge from VBH for intervention procedures to be initiated.

Routine care and study activities are described in Table 1. The intervention incorporated TAU with the addition of (1) a treatment partner contracting and psychoeducation session and (2) text message reminders of clinic appointments. Intervention development was informed by focus group and in-depth interview work (published elsewhere), where we found there was a need for caregiving relationships to be negotiated to protect he MHSU’s autonomy [17].

**Table 1:** Routine care, timelines and corresponding study activities

Stage in MHSU care	MHSU care activity	Corresponding research activity	
	Routine care	Treatment as usual = routine care PLUS	Intervention = treatment as usual PLUS
In pre-discharge ward	The MHSU receives standard pre-discharge care. This includes Clinical review Finalizing of treatment plan Psychosocial rehabilitation programmes Individual and group psychoeducation Discharge planning by the clinical team, encompassing identification of which CHC the MHSU will be discharged to and when, as well as arrangement of post-discharge psychosocial support for the MHSU	1. We obtained informed consent from eligible patients 2. We conducted recruitment activities including diagnostic and baseline measures as described in the text 3. We randomized recruited participants to receive either treatment as usual or the intervention 4. We informed participants which group they had been randomized to	5. Participants who had been randomized to receive the intervention selected a treatment partner as described in the text 6. These selected treatment partners were contacted telephonically, consented and a date was set for the psychoeducation and contracting session 7. The psychoeducation and contracting session was conducted at Valkenberg Hospital for the participant and treatment partner pair
On day of discharge	The MHSU discharged from inpatient care with referral letter detailing course of admission, diagnosis, treatment plan, review CHC and date	8. Participants were enrolled onto the text message platform	9. The first text message was sent to the participant/ treatment partner pair containing details of first clinic appointment as per discharge treatment plan 10. Fieldworker handsets received updated participant review schedules
1 week before first clinic appointment	No activity	No activity	11. A text message reminder was sent to the participant/treatment partner pair containing same details as first message
At first clinic appointment	The MHSU is reviewed by the mental health nurse or psychiatry registrar at the psychiatry clinic of CHC Medication is renewed or modified A follow up appointment is issued The MHSU’s CHC clinical record clinical record updated to indicate Attendance for review Clinical status Medication review and prescription Next scheduled appointment date The MHSU collects his or her medication and leaves the CHC	No additional activity	12. On arrival, mental health nurse checks the participant as present 13. The mental health nurse asks the participant how many days of medication have been missed since discharge from hospital and enters this information on the fieldworker handset 14. The mental health nurse enters the next scheduled clinic review appointment date onto the text message platform via the fieldworker handset 15. The participant/treatment partner pair immediately received a text message notification indicating the next appointment date with the name of the CHC as in the initial text message
After the first clinic appointment	The MHSU is reviewed on the scheduled review date and the process described above at first clinic appointment is repeated for subsequent visits	No additional activity	16. The participant/treatment partner pair receives a text message reminder of the next appointment date one week before that appointment 17. The process described at steps 12-16 above is repeated for three visits in total
3 months after discharge	Routine clinical care is continued	18. All participants, accompanied by their caregivers for the TAU group or treatment partners for the intervention group, returned at 3 months after discharge for clinical and review and qualitative interviews as described in the text	
9 months after discharge		19. Any readmissions were noted for all participants were noted via Clinicom	

The treatment partner contracting and psychoeducation session was conducted with the intervention pair at VBH on the agreed-upon date. Psychoeducation focused on the participant’s diagnosis and was based on the national institute for mental health and the VBH psychoeducation guidelines, with input from specialist psychiatrists at VBH [18]. Psychoeducation addressed mental health literacy needs uncovered during our formative work and included psychiatry clinic follow-up processes. An individualized participant/treatment partner relationship was negotiated and agreed upon, detailing a communication and adherence-support approach.

Upon discharge, participant information was loaded onto a text message system using one enrolment handset. Fifteen fieldworker handsets were programmed for use by the nurses. Once enrolled, the fieldworker handsets were remotely populated with the relevant participant information. Once discharged, the intervention pair received a text message, indicating the appointment clinic and date. Another text message was sent 1 week before the appointment. TAU participants received no text messages. When participants arrived at the clinic the nurse used the handsets to mark the participant’s attendance, and to schedule a follow-up appointment on the system, which then sent similar notifications to those sent upon discharge. Nurses scheduled 2 follow-up appointments in total.

#### Assessment measures

Participants underwent a structured clinical interview for diagnosis of axis-I disorders (SCID-I) to confirm diagnosis, which included a global assessment of function scale (GAF) and clinical global impressions scale (CGI). We administered the positive and negative syndrome scale (PANSS) to measure severity of psychotic symptoms. These scales have been widely used locally in genetics and treatment studies [3, 19–22]. The Camberwell assessment of needs scale (CAN) was used to measure met and unmet needs [23]. The EUROQOL was used to measure functional level, and the medication adherence rating scale (MARS) was used as a measure of medication adherence [24, 25].

We used the text message system to track intervention group attendance at first clinic visit. The attendance of both groups was additionally captured retrospectively from the attendance registers at the clinics. All participants needed to return for a follow-up study visit 3-months from enrolment. The MARS, PANSS, CAN and EUROQUOL were re-administered at this visit, following which a semi-structured interview was conducted with all participants. Participants were asked about their diagnosis and treatment, their adherence behaviour and about their clinic experience. TAU group were asked about the standard pre-discharge psychoeducation, and the intervention group about the psychoeducation session. Caregivers and treatment partners provided feedback about the experience of their adherence support role, and on participants’ adherence behaviour. Intervention pairs provided feedback on the components of the intervention. These audio-recorded interviews were conducted in the language of respondents’ choice, in the presence of a translator and transcribed before undergoing thematic analysis.

At 9 months, readmissions were noted via Clinicom, a secure online platform used to track health service users accessing public health services in Cape Town, which maintains detailed notes regarding assessments, admissions, and treatment (see Additional file 1).

We expected that the intervention would be acceptable and feasible, and result in increased first clinic visits and reduced 9-month readmissions. We expected an improvement in medication adherence and quality of life, a reduction of needs and symptomatic improvement.

#### Statistical analysis

Primary outcomes were intervention acceptability and feasibility. Efficacy outcomes, which were secondary, were adherence to first clinic visit; relapse, defined as readmission to hospital; medication adherence; quality of life and symptomatic relief. Data for clinic visits and readmission were complete. The efficacy outcomes had incomplete outcome data as only 34 participants attended the study visit at 3 months. We report all effect measures as risk ratios or mean differences with their corresponding 95% confidence intervals. A two-tailed significance level of 5% was used throughout and analyses were conducted using Stata version 13.

## Results

The participant flow diagram is available as an additional figure (see Additional file 2). Seventy-seven participants were randomized, 42 to the intervention and 35 to TAU. Efficacy outcomes were analyzed at the end of the 2-year recruitment period (n = 77). With the significant participant attrition at 3-month study follow up, we calculated that a sample size of 520 would be required to demonstrate a moderate effect.

Sample demographic characteristics are presented in Table 2.

**Table 2:** Baseline variables

	Total sample^a^ (N = 77)		Intervention (N = 42)		TAU (N = 35)		Statistic (df)	p value
	Mean	(SD)	Mean	(SD)	Mean	(SD)		
Participant characteristics								
Age	35.5	(10.2)	35.3	10.9	35.8	9.5	t = − 0.35 (75)	0.726
	N	(%)	N	(%)	N	(%)		
Sex							χ^2^ = 1.03 (1)	0.311
Male	55	71.4	28	66.6	27	77.1		
Female	22	28.6	14	33.3	8	22.9		
Ethnicity							χ^2^ = 0.51 (2)	0.774
Coloured	47	66.2	26	66.7	21	65.6		
Black	18	25.3	9	23.1	9	23.1		
Other	6	8.5	4	10.3	2	6.3		
Marital status							χ^2^ = 0.29 (1)	0.591
Never married	51	71.8	27	69.2	24	75.0		
Ever married	20	28.2	12	30.8	8	25.0		
Highest level of education							χ^2^ = 0.15 (2)	0.930
Grade 7 or less	12	17.7	6	16.7	6	18.7		
Grades 8 to 11	42	61.8	22	61.1	20	62.5		
Grade 12	14	20.6	8	22.2	6	18.8		
6 month employment							χ^2^ = 0.31 (1)	0.579
Unemployed	47	69.1	28	71.8	19	65.5		
Employed	21	30.9	11	28.2	10	34.5		
Diagnosis								0.604
Schizophrenia Spectrum	62	80.5	32	76.2	30	85.7		
Bipolar mood disorder	11	14.3	7	16.7	4	11.4		
Substance induced psychotic disorder	4	5.2	3	7.1	1	2.9		
Substance use							χ^2^ = 0.18 (1)	0.671
Lifetime substance use disorder	31	40,3	16	38.1	15	42.9		
Antipsychotic								
First generation	50	64.9	26	61.9	24	68.6	χ^2^ = 0.37 (1)	0.542
Second generation	19	24.7	12	28.6	7	20.0	χ^2^ = 0.75 (1)	0.385
Long acting injectable	22	28.6	10	23.8	12	34.29	χ^2^ = 1.03 (1)	0.311
Baseline measures								
PANSS subscales								
Positive	15.4	6.5	15.6	6.9	15.2	6.2	t = 0.06 (73)	0.951
Negative	14.4	4.7	13.8	4.8	15.1	4.5	t = − 1.47 (73)	0.015
General	26.8	7.5	26.5	7.9	27.1	7.2	t = − 0.44 (73)	0.663
Total	56.6	15.9	55.9	17.1	57.4	14.5	t = − 0.42 (73)	0.676
CGI	3.5	1	3.4	1	3.7	1	t = − 0.96 (68)	0.340
GAF	48.8	10.1	49.9	10.8	47.6	9.4	z = 1.170	0.242
CAN unmet needs	4.1	2.98	3.6	2.97	4.7	2.94	z = − 1.705	0.088
EUROQUEL VAS	8.4	20.7	84	21.2	84.8	20.4	z = − 0.200	0.842
MARS	5.9	1.88	5.8	1.87	6.0	1.93	t = − 0.36 (71)	0.723

^a^Baseline variables with missing data included: marital status: n = 6, ethnicity: n = 6, HLOE: n = 9, employment = 9, PANSS: n = 2, CGI: n = 7, GAF score: n = 14, CAN: n = 6, EUROQUEL VAS: n = 3, MARS: n = 4

Treatment partners included family members, partners and friends. At 3-month review, intervention participants understood their diagnosis better than TAU participants. Understanding of medication regimen and reported treatment adherence were similarly low in both groups. The psychoeducation session was seen to be superior to standard pre-discharge psychoeducation. Treatment partners showed a better knowledge of diagnosis. Intervention pairs felt that a psychoeducation follow-up would be valuable in the long-term to reinforce knowledge.

Some participants (41.2%) did not receive the text messages, either due to changed cellphone numbers or due to misplacement or theft of cellphones. Those who received the text messages all found them helpful. The nurses found the refreshing of handsets burdensome and challenging in spite of reinforcement training. Three of the fieldworker handsets were lost through theft.

All participants found the clinic easy to access and navigate. More treatment partners than caregivers found the clinic helpful in supporting adherence. The experiences of being a treatment partner and a caregiver were similar, with half in each group reporting a positive experience and the other half experiencing it as challenging [26].

The intervention was acceptable. The psychoeducation and the treatment partner components were feasible while there were significant challenges with the text message system.

Efficacy outcome data is represented in Table 3. TAU participants were more likely to miss their first clinic visit and to relapse in the 9 months following discharge. At 3 month review, TAU participants were more likely to show worsening PANNS scores, while GAF scores showed better improvement amongst intervention participants. CGI scores suggested a symptom improvement for the intervention group while the GAF scores of both groups suggested general improvement in functioning. There was a trend towards an increase in unmet needs in the TAU group vs a decline in the intervention group, while met needs were seen to increase in both groups.

**Table 3:** Efficacy outcomes

Outcome	n	%	Risk ratio (ITT)		p value	95% CI
			Unadjusted (n = 77)	Adjusted^a^ (n = 77)		
Intention-to-treat analysis (ITT): non-adherence to first clinic visit, re-admission over 9 months						
Non-adherence to first clinic appointment						
Intervention (n = 42)	14	33.3	0.72	0.79	0.419	0.44 to 1.39
Treatment as usual (n = 35)	16	45.7	–	–	–	–
Any re-admission over 9 months						
Intervention (n = 42)	5	11.9	0.83	0.86	0.713	0.39 to 1.87
Treatment as usual (n = 35)	5	14.3	–	–	–	–

Outcome	Complete case analysis^c^ (intervention vs. TAU)				ITT (intervention vs. TAU)			
	Mean difference^d^		p value	95% CI	Mean difference^e^ (n = 77)		p value	95% CI
	Unadjusted	Adjusted			Unadjusted	Adjusted		
Complete case and intention to treat analysis (ITT) of other efficacy outcomes at 3 months								
PANSS score								
Total score	− 9.4	− 14.7	0.052	− 29.71 to 0.16	− 13.4	− 13.1	0.062	− 27.00 to 0.73
Positive subscale	− 3.8	− 6.4	0.011	− 11.20 to 1.60	− 5.6	− 5.4	0.060	− 11.16 to 0.25
Negative subscale	− 2.6	−4.4	0.059	− 8.99 to 0.18	− 3.5	− 3.5	0.078	− 7.52 to 0.43
General subscale	− 2.8	− 3.9	0.350	− 12.6 1 to 4.68	− 4.4	− 4.2	0.248	− 11.67 to 3.19
MARS	− 0.21	− 0.75	0.425	− 2.68 to 1.17	0.36	0.49	0.603	− 1.44 to 2.43
CGI	− 0.8	− 0.58	0.346	− 1.84 to 0.67	–	–	–	–
GAF	7.5	4.1	0.440	− 6.90 to 15.17	–	–	–	–
CAN					–	–	–	–
Total needs	–	–	–	–	–	–	–	–
Unmet needs	− 3.2	− 3.6	0.029	− 6.74 to 0.49	–	–	–	–
Met needs	–	–	–	–	–	–	–	–
EUROQUEL-VAS	16.1	15.2	0.124	− 4.59 to 34.99	–	–	–	–

^a^Log-Poisson regression model with robust variance estimation, ITT; adjusted for age, sex, substance use disorder, baseline scores of PANSS total score, GAF score, MARS, EUROQUEL VAS scale, CAN unmet needs score

^b^Log-Poisson regression model with robust variance estimation, ITT; sex dropped from model, adjusted for adjusted for age, substance use disorder, baseline scores of PANSS total score, GAF score, MARS, EUROQUEL VAS scale, CAN unmet needs score. Baseline data were imputed

^c^Multiple linear regression models adjusted for age, sex, substance use disorders, baseline scores of PANSS total score, GAF, EUROQUEL, MARS, CAN unmet needs. Models with violation of linear regression assumptions omitted

^d^Sample size varied due to list-wise deletion

^e^Only models for which missing data < 55% are reported

## Discussion

We found that (1) The treatment-partner and psychoeducation components were acceptable and feasible; (2) The text message component was acceptable but not feasible in its current form; and (3) Efficacy outcomes favoured the intervention but did not reach statistical significance.

The acceptability and feasibility of this intervention is consistent with prior literature [8]. Our approach was in line with previous caregiver focused interventions, which have targeted mental health literacy on the experience of caregiving. Family psychoeducation interventions have reduced relapse in people living with severe mental illness, while helping to meet caregiver needs [27, 28]. While it has been more difficult to change coping styles and reduce caregiver burden, short term interventions have improved caregivers’ knowledge and attitudes towards MHSU and mental illness [29, 30].

Brief psychoeducation has reduced relapse in the medium term and promoted medication adherence in the short term [31]. Improved clinical outcomes have included reduced relapse and improvement in treatment adherence [32]. Many of our participants reported inadequate and inconsistent current psychoeducation approaches within routine MHSU discharge processes, potentially alluding to inadequate discharge planning. Good discharge planning may be affected by the quality of the therapeutic alliance, which in turn is often impacted by the clinician’s clinical experience [33–36].

While text messaging is a promising and acceptable tool to aid medication and clinic adherence in mental illness, the evidence base remains inconclusive [14, 37, 38]. Our formative work found support for text message prompts [17]. Some challenges encountered during the trial however, included participant factors such as changing numbers and loss of handsets, impacting negatively on the utility of text messaging, as did fieldworker factors including software challenges and loss of handsets. Socioeconomic factors and the complexity of the software thus impacted negatively on the utility of text message prompts in our setting.

In conclusion, a treatment-partner psychoeducation intervention is acceptable and feasible in a LMIC setting. Psychoeducation must be tailored to the needs of the specific population targeted, and take into account the need for ongoing reinforcement. M-health may have potential to improve adherence, but text message prompts may be problematic in some LMIC settings such as ours at present. Such additional components of such interventions must be tailored to the local context. The assessment of efficacy for such an intervention requires appropriately powered studies.

## Limitations

- Sample size was insufficiently powered for the efficacy analyses. Our primary focus of was on acceptability and feasibility. The work done here allows a power analysis for future efficacy research using larger samples.
- The mental health nurses experienced challenges interacting with the text message system. This compromised appointment checking and rescheduling.

## Additional files

**Additional file 1.** Time frames and associated instruments.

**Additional file 2.** CONSORT Study flow chart.

## Abbreviations

LMIC: low- and middle-income countries
M-health: mobile health
MHSU: mental health service user
CHC: community health centre
VBH: Valkenberg hospital
TAU: treatment as usual
SCID-1: structured clinical interview for diagnosis of axis-I disorders
GAF: global assessment of function scale
CGI: clinical global impressions
PANSS: positive and negative syndrome scale
CAN: Camberwell assessment of needs scale
MARS: medication adherence rating scale
SD: standard deviation
LOCF: last observation carried forward
MAR: missing at random
MICE: multiple imputation with chained equations
VBH: Valkenberg hospital
ITT: intention to treat analysis
