# Supplementary material for: Improving adherence in mental health service users with severe mental illness in South Africa: a pilot randomized controlled trial of a treatment partner and text message intervention vs. treatment as usual
Source: BMC Res Notes. 2017 Nov 9;10:584. doi: 10.1186/s13104-017-2915-z (PMC5679373; doi:10.1186/s13104-017-2915-z)
Supplement: Supplementary file 1 — Additional file 1. Time frames and associated instruments. [file 13104_2017_2915_MOESM1_ESM.docx]

**Additional file 1**

| **Initiation** | **3 Month Follow Up** | **9 month** |
| --- | --- | --- |
| 1. Participant selection as per inclusion criteria 2. Randomisation 3. Consent and contract 4. Baseline instruments   SCID^1^  CGI^2^  GAF^3^  MARS^4^  CANS^4^  PANSS^6^  EUROQUOL | 1. Qualitative review:    1. MHSU perspective,    2. Treatment partner or caregiver perspective. 2. Review appointment adherence 3. Determine and record re-admissions via Clinicom 4. Efficacy measures    1. MARS, CGI, GAF, PANSS and EUROQOL | Determine and record re-admissions via Clinicom |

^1^Structured Clinical Interview for DSM disorders; ^2^Clinical Global Impression Scale; ^3^Global Assessment of Function Scale; ^4^Medication Adherence Rating Scale; ^5^Camberwell Assessment of Needs Scale; ^6^Postive and Negative Syndrome Scale
