# Supplementary material for: Improving adherence in mental health service users with severe mental illness in South Africa: a pilot randomized controlled trial of a treatment partner and text message intervention vs. treatment as usual
Source: BMC Res Notes. 2017 Nov 9;10:584. doi: 10.1186/s13104-017-2915-z (PMC5679373; doi:10.1186/s13104-017-2915-z)
Supplement: Supplementary file 2 — Additional file 2. CONSORT Study flow chart. [file 13104_2017_2915_MOESM2_ESM.doc]

**Additional file 2**

**Allocation**

**Analysis**

**Follow-Up**

**Enrolment**

Assessed for eligibility (n=111)

Excluded (n=34)

- Declined to participate (n=29)
- Other reasons (n=4)

Analysed (n=42)

Lost to follow-up (n=20)

- Could not be tracked down (n=18)
- Did not arrive for scheduled follow up (n=2)

Allocated to receive intervention (n=42)

- Received treatment partner and mobile health intervention (n=37)
- Withdrew at baseline (n=5)

Lost to follow-up (n=18)

- Could not be tracked down (n=10)
- Did not arrive for scheduled follow up (n=7)
- Refused to return for follow-up (n=1)

Allocated to treatment as usual (n= 35)

- Discharged to follow up at local CHC as per standard practice

Analysed (n= 35)

Randomized (n=77)
